# Venetoclax Induces Cardiotoxicity through Modulation of Oxidative-Stress-Mediated Cardiac Inflammation and Apoptosis via NF-κB and BCL-2 Pathway

**DOI:** 10.3390/ijms23116260

**Published:** 2022-06-02

**Authors:** Abdullah F. AlAsmari, Adel Alghamdi, Nemat Ali, Muath A. Almeaikl, Hassan M. Hakami, Meshal K. Alyousef, Mohammed AlSwayyed, Metab Alharbi, Faleh Alqahtani, Fawaz Alasmari, Nasser Alsaleh

**Affiliations:** 1 Department of Pharmacology and Toxicology, College of Pharmacy, King Saud University, Riyadh 11451, Saudi Arabia; 441105941@student.ksu.edu.sa (A.A.); nali1@ksu.edu.sa (N.A.); ph.muatham@gmail.com (M.A.A.); 437103103@student.ksu.edu.sa (H.M.H.); 437104079@student.ksu.edu.sa (M.K.A.); mesalharbi@ksu.edu.sa (M.A.); afaleh@ksu.edu.sa (F.A.); ffalasmari@ksu.edu.sa (F.A.); nbalsaleh@ksu.edu.sa (N.A.); 2 Department of Pathology, College of Medicine, King Saud University, Riyadh 11451, Saudi Arabia; malswayyed@ksu.edu.sa

**Keywords:** cardiotoxicity, apoptosis, venetoclax, inflammation, oxidative stress

## Abstract

Cardiovascular damage induced by anticancer therapy has become the main health problem after tumor elimination. Venetoclax (VTX) is a promising novel agent that has been proven to have a high efficacy in multiple hematological diseases, especially acute myeloid leukemia (AML) and chronic lymphocytic leukemia (CLL). Considering its mechanism of action, the possibility that VTX may cause cardiotoxicity cannot be ruled out. Therefore, this study was designed to investigate the toxic effect of VTX on the heart. Male Sprague-Dawley rats were randomly divided into three groups: control, low-dose VTX (50 mg/kg via oral gavage), and high-dose VTX (100 mg/kg via oral gavage). After 21 days, blood and tissue samples were collected for histopathological, biochemical, gene, and protein analyses. We demonstrated that VTX treatment resulted in cardiac damages as evidenced by major changes in histopathology and markedly elevated cardiac enzymes and hypertrophic genes markers. Moreover, we observed a drastic increase in oxidative stress, as well as inflammatory and apoptotic markers, with a remarkable decline in the levels of *Bcl-2*. To the best of our knowledge, this study is the first to report the cardiotoxic effect of VTX. Further experiments and future studies are strongly needed to comprehensively understand the cardiotoxic effect of VTX.

## 1. Introduction

Drug-induced toxicity effects on cardiovascular function or tissues are not only a serious health issue, but they are often detected after the introduction of the drug in clinical practice [1]. This reflects that these high-risk cardiovascular events are either not detected in earlier clinical trials, or those that arise when drugs are administrated for long periods of time to larger patient population are not considered to be biologically significant [1]. Such a high incidence of cardiovascular adverse drug reactions in late-stage clinical development can lead to additional pre- and/or postapproval monitoring, prescribing restrictions, dose-limiting toxicity, or ultimately drug discontinuation or withdrawal [1]. Importantly, these drug-induced cardiovascular toxicity events are considered the primary cause of drug withdrawal from the market [1,2].

The list of anticancer therapy drugs that can potentially cause cardiotoxicity-related adverse effects is growing [3]. This raises an important issue in cancer treatment, as it can influence the mortality and morbidity of patients with cancer by causing a delay or discontinuation of chemotherapy [4]. Over the past two decades, anticancer therapy has resulted in remarkable advances in both the survival rate and quality of life of cancer patients [5]. Although anticancer agents have shown efficacy against different types of tumors, many reports have demonstrated its cardiotoxic effect [5,6]. Cardiotoxicity represents the most feared adverse reaction to chemotherapy, with a growing incidence of up to 30% of patients receiving chemotherapy developing a cardiovascular side effect during their life, which leads to an increase in morbidity and mortality [7,8,9]. It is worthy to note that the cardiotoxic side effects induced by long-term use of anticancer therapy has been one of greatest challenges after tumor elimination [10]. Therefore, it is essential for oncologists to know the cardiotoxicity profile of newer agents and determine the etiology and most appropriate management of these various effects so they can consider the risks and benefits of eliminating the tumor and preservation of cardiac function [3,11].

Venetoclax (VTX), or ABT-199, is a promising novel agent that has been proven to have a high efficacy in multiple hematological diseases, especially chronic lymphocytic leukemia (CLL) and acute myeloid leukemia (AML) [12]. Of all newly diagnosed cancer patients, 6.5% have blood cancers such as acute leukemia, non-Hodgkin lymphoma, Hodgkin lymphoma, and multiple myeloma [12]. Apoptosis resistance of CLL cells is mediated through Bcl-2 overexpression [13]. When Bcl-2 is overexpressed, this leads to tumor formation, as in CLL and follicular lymphoma, inappropriate cell survival, and chemotherapy resistance [14,15]. Apoptosis can be initiated by BH3-only proteins in response to significant stresses, such as genetic damage [14]. Thus, higher levels of Bcl-2 overcome the BH3-only proteins and result in evading apoptosis [16]. VTX targets the BH3 domain of Bcl-2 as a BH3 mimetic (Bcl-2 inhibitor) that can restore apoptosis in malignant cells [17]. Indeed, VTX have a high affinity to the BH3-binding groove of Bcl-2, which leads to displacement of the bounded proapoptotic BH3-only proteins [18]. Therefore, these free BH3-only proteins lead to displacement and activation of apoptotic effectors (e.g., Bax) from binding to antiapoptotic members [18]. Eventually, VTX induces the release of proapoptotic from Bcl-2 and restores apoptosis in tumor cells [18]. In the clinical sitting, it has been reported that VTX caused cardiomyopathy and cardiac arrhythmia [19,20,21]. Therefore, we hypothesized that VTX treatment can cause toxic effects to the heart. It is well known that the Bcl-2 family of proteins are essential regulators of apoptosis [22]. Moreover, it has been reported that inhibition of Bcl-2 can lead to apoptosis in different organs, especially the heart, and consequently resulting in organ toxicity [21,23]. Therefore, in the current study, we investigated the toxic effects of VTX on the heart, and examined the signal and molecular mechanism of its toxicity.

## 2. Results

### 2.1. Effect of VTX Treatment on Cardiac Enzymes

**Figure 1:**
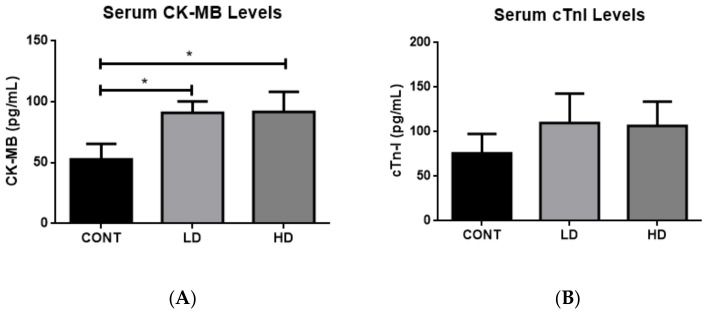
Serum levels of cardiac enzymes. Sera from different groups were analyzed to measure CK-MB and cTnI levels (**A**,**B**, respectively). Data are presented as mean ± SD (*n* = 5). * *p* < 0.05. Cont, control; LDV, low-dose venetoclax (50 mg/kg); HDV, high-dose venetoclax (100 mg/kg); CK-MB, creatine kinase-MB; cTn-I, troponin I cardiac muscle.

It has been demonstrated that cardiac enzymes increase in blood when there are some injuries or damage to the heart. Therefore, we measured the serum level of CK-MB and cardiac troponin I (cTn-I) in rats after 21 days of VTX treatment. Surprisingly, we found that the treatment of VTX increased the serum levels of both CK-MB (Figure 1A) and cTn-I (Figure 1B), but the increase in cTn-I was not significant. These results indicated that VTX treatment induced cardiac damage.

### 2.2. VTX-Induced Cardiac Hypertrophy

**Figure 2:**
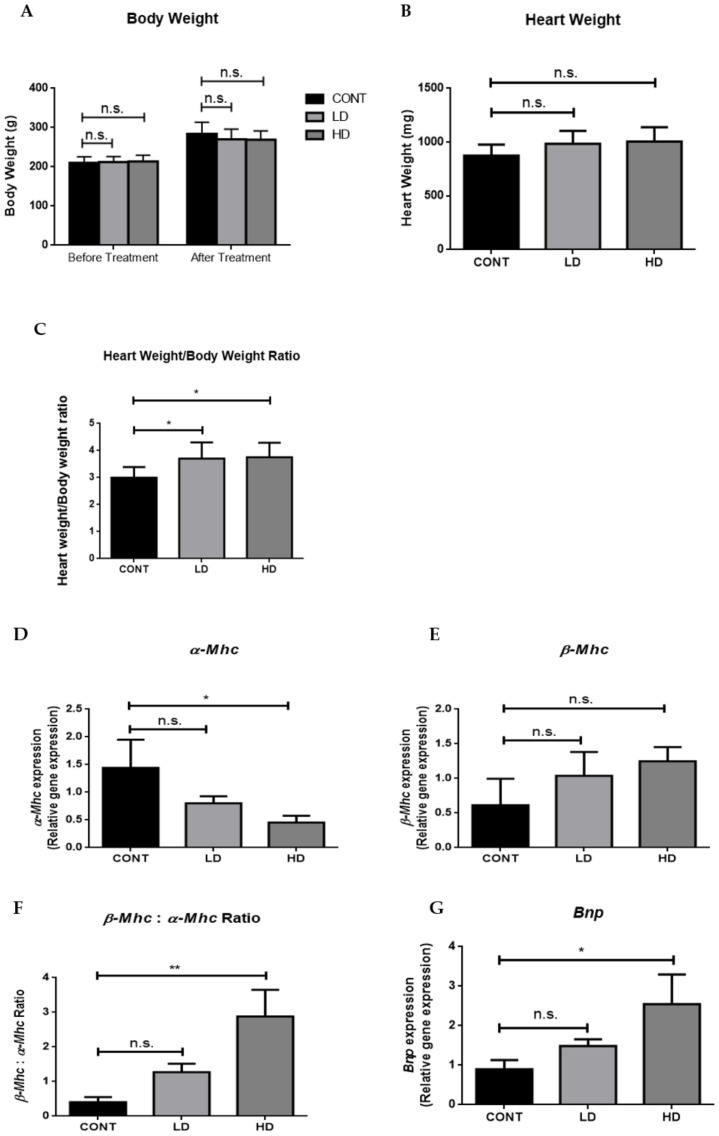
VTX-induced cardiac hypertrophy. (**A**) Body weight; (**B**) heart weight; and (**C**) heart weight to body weight ratio. Data are presented as mean ± SD. * *p* < 0.05; n.s., no significant changes were observed (*p* > 0.05). (**D**–**G**) mRNA levels of (**D**) α-*Mhc*, (**E**) β-*Mhc*, (**F**) β-*Mhc* to α-*Mhc* ratio, and (**G**) *Bnp* were measured using RT-PCR. Data are presented as mean ± SD (*n* = 5). * *p* < 0.05, ** *p* < 0.01; n.s., no significant changes were observed (*p* > 0.05). Data were normalized to β-actin as a housekeeping gene and one-way analysis of variance (ANOVA), followed by Tukey–Kramer multiple-comparisons tests. Cont, control; LD, low-dose venetoclax (50 mg/kg); HD, high-dose venetoclax (100 mg/kg); *α-Mhc*, alpha myosin heavy chain; *α-Mhc,* beta myosin heavy chain; *Bnp*, brain natriuretic peptide.

Numerous studies have shown that cardiac hypertrophy can be a maladaptive process in response to intrinsic or extrinsic stresses. Therefore, we measured the body weight (BW), heart weight (HW) and heart weight/body weight ratio (HW/BW), which we used as an indicator of cardiac hypertrophy, and measured the expression of three genes that are known to be involved in this process. As shown in Figure 2A,B, no significant changes were observed in the BW or HW between all groups. However, a significant increase in the HW/BW ratio was observed in VTX-treated groups compared to the control group (Figure 2D). Furthermore, we found that the gene-expression level of *α-Mhc* decreased in VTX-treated groups compared to the control group, while the gene-expression levels of β-*Mhc* and the β-*Mhc* to α-*Mhc* ratio increased significantly in the VTX-treated groups compared to the control group (Figure 2A–C, respectively). Furthermore, the gene-expression level of *Bnp* was substantially increased in the VTX-treated groups (Figure 2D). Overall, these results indicated that VTX treatment induced cardiac hypertrophy and damage.

### 2.3. Effects of VTX on Cardiac Architecture

**Figure 3:**
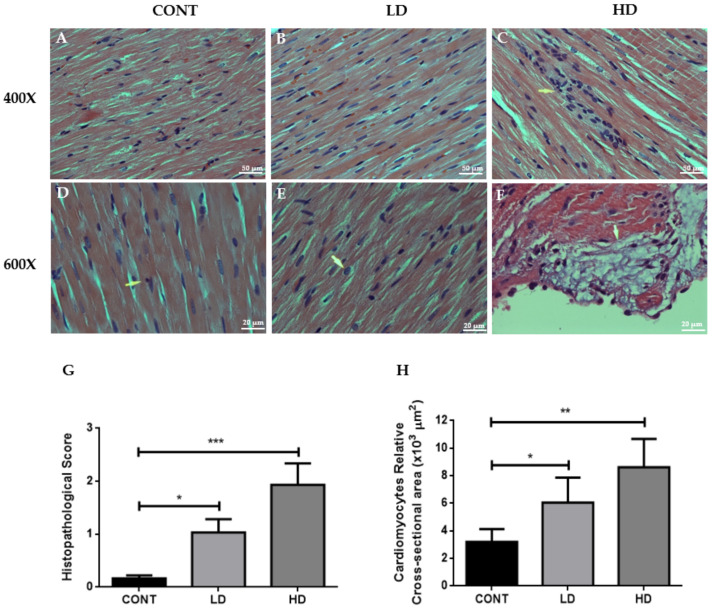
Light micrographs of cardiac tissues using H&E stain. (**A**,**D**) Represents the regular, parallel, and branching striated myocardial fibers with cross-striation and regular nuclei with no evidence of inflammation or necrosis. (**B**,**E**) Myocardium section obtained from low-dose VTX shows normal appearance of the myocardial fibers, but with slightly nuclear enlargement. (**C**,**F**) Represents heart section from rats treated with a high dose of VTX, and shows the presence of a focus of myocardial damage associated with chronic inflammatory reaction (arrowhead). Images (**A**–**C**) obtained at 400× magnification, while images (**D**–**F**) were obtained at 600× magnification (scale bar = 50 µm and 20 µm, respectively). (**G**) Histopathological score. (**H**) Cardiomyocytes’ cross-sectional area. Data are presented as mean ± SD (*n* = 3). * *p* < 0.05, ** *p* < 0.01, *** *p* < 0.001. Cont, control; LD, low-dose venetoclax (50 mg/kg); HD, high-dose venetoclax (100 mg/kg).

It has been demonstrated that changes in and damage to cardiac cell morphology correlate with many diseases and toxicity. Therefore, we examined the heart architecture of rats after 21 days of VTX treatment. We observed a normal and parallel myocardial fiber with cross-striation and regular nuclei in the myocardium sections obtained from the control group (Figure 3A,D). Sections of myocardium obtained from the second group, which received a low dose of VTX, also showed a normal appearance of the myocardial fibers, but with minimal nuclear enlargement (Figure 3B,E). Interestingly, heart sections from the third group, which received a high dose of VTX, showed the presence of a focus of myocardial damage associated with chronic inflammatory reaction (arrowhead) (Figure 3C,F). Figure 3F shows the arrowhead area at 600× magnification, and indicates the presence of a focus of subendocardial myxoid degeneration. This feature is not well understood, but could indicate myocardial damage in this area. Furthermore, we observed that VTX treatment increased the cardiomyocytes’ cross-sectional area considerably in a dose-dependent pattern (Figure 3H). Taken together, VTX treatment induced morphological changes in cardiac tissues.

### 2.4. VTX-Induced Apoptosis in the Heart

**Figure 4:**
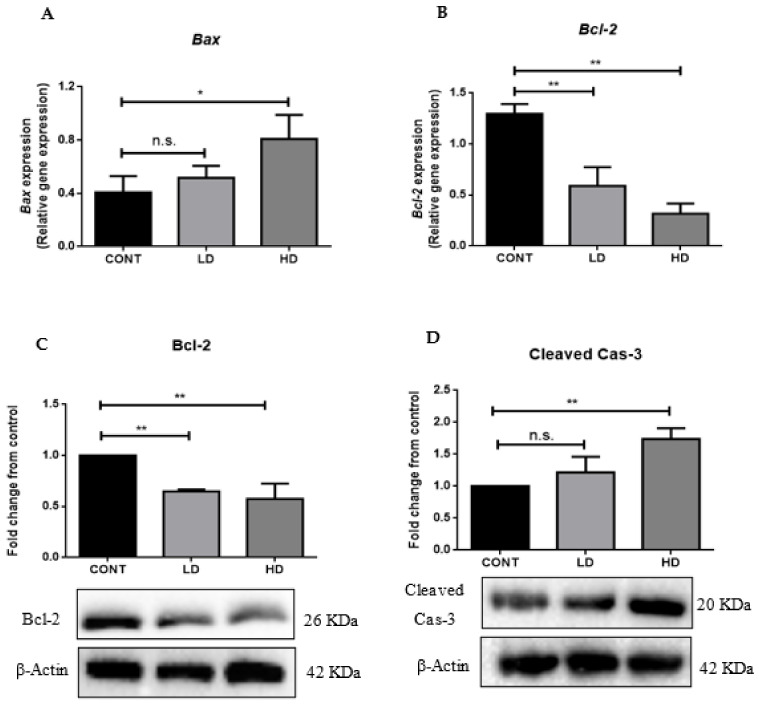
VTX-induced apoptosis in the heart. The mRNA levels of *Bax* (**A**) and *Bcl-2* (**B**) were measured using RT-PCR. Data were normalized to β-actin as a housekeeping gene and one-way analysis of variance (ANOVA), followed by Tukey–Kramer multiple-comparisons tests. (**C**) Representative Western blot analysis of Bcl-2 protein levels. (**D**) Representative Western blot analysis of Cleaved Cas-3 protein levels. Data are presented as mean ± SD (*n* = 5). * *p* < 0.05, ** *p* < 0.01; n.s., no significant changes were observed (*p* > 0.05). Cont, control; LD, low-dose venetoclax (50 mg/kg); HD, high-dose venetoclax (100 mg/kg); Bax, Bcl-2 associated X; Bcl-2, B-cell lymphoma 2; Cleaved Cas-3, cystinyl aspartate-specific proteases 3; β-actin, beta actin.

Several studies have linked myocardial dysfunction and toxicity with apoptosis. Therefore, we measured the gene and protein expressions of multiple apoptotic markers to examine the induction of apoptosis in heart tissues. We found that 21 days of VTX treatment induced the gene expression of *Bax* compared to the control group (Figure 4A). Moreover, a significant decline was observed in the *Bcl-2* gene and protein expressions in VTX-treated rats compared to the control group (Figure 4B,C, respectively). Nonetheless, a Western blot analysis revealed that treatment with a high dose of VTX resulted in a notable rise in the protein levels of cleaved caspase-3 (Cleaved Cas-3) compared to the control group (Figure 4D).

### 2.5. VTX-Induced Oxidative Stress and Inflammation in the Heart

**Figure 5:**
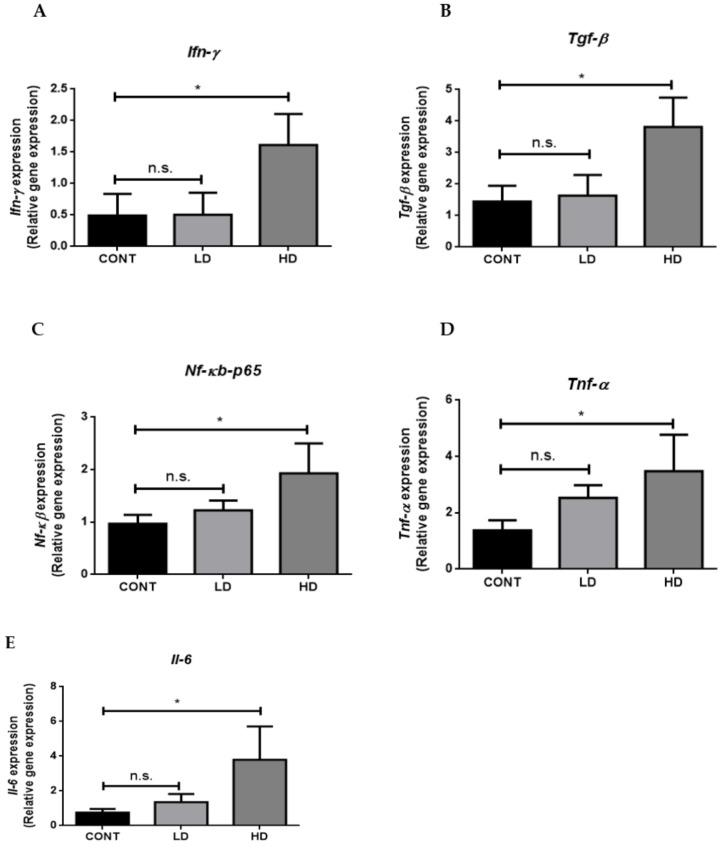

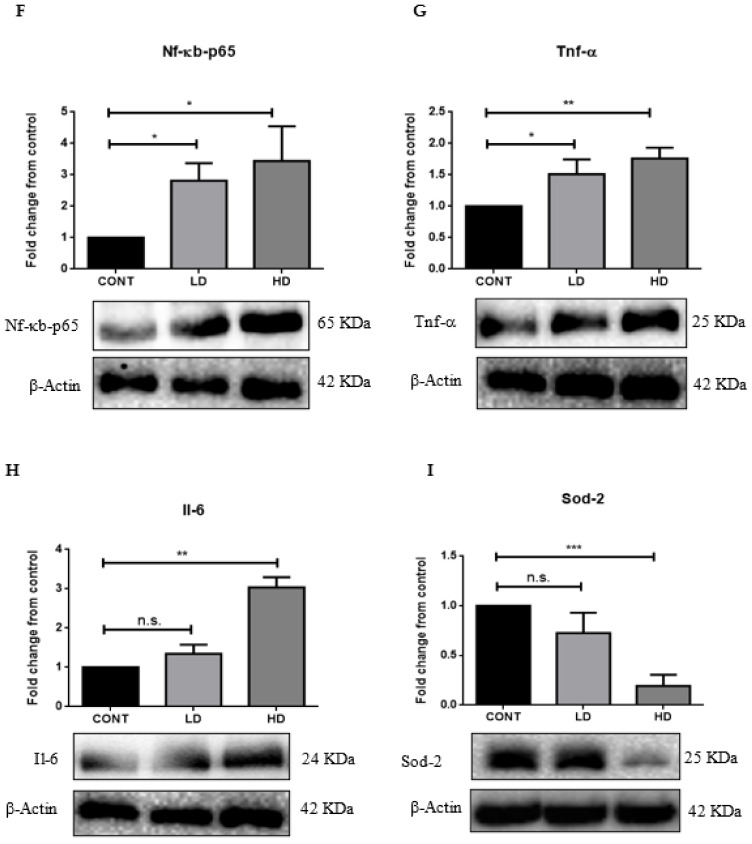
VTX-induced oxidative stress and inflammation in the heart. (**A**–**E**) The mRNA levels of *Ifn-γ*, *Tgf-β*, *Nf-κb-p-65*, *Tnf-α*, and *Il-6* were measured using RT-PCR. Data were normalized to β-actin as a housekeeping gene and one-way analysis of variance (ANOVA), followed by Tukey–Kramer multiple-comparisons tests. (**F**–**I**) Representative Western blot analysis of protein levels of Nf-κb-p-65, Tnf-α, Il-6, and Sod-2. Data are presented as mean ± SD (*n* = 5). * *p* < 0.05, ** *p* < 0.01, *** *p* < 0.001; n.s., no significant changes were observed (*p* > 0.05). Cont, control; LD, low-dose venetoclax (50 mg/kg); HD, high-dose venetoclax (100 mg/kg); Ifn-γ, interferon gamma; Tgf-β, transforming growth factor beta; Nf-κb-p-65, nuclear factor kappa-B; Tnf-α, tumor necrosis factor alpha; Il-6, interleukin-6; Sod-2, superoxide dismutase-2; β-actin, beta actin.

It has been demonstrated that oxidative stress and inflammation are the main mechanisms that induce cardiac toxicity. Therefore, we measured the gene and protein expressions of different inflammatory and oxidative stress markers. We found that the gene-expression levels of *Ifn-γ* (Figure 5A) and *Tgf-β* (Figure 5B) sharply rose in the VTX treatment group compared to the control group. Furthermore, the gene and protein levels of *Nf-κb-p-65* in VTX-treated rats were significantly increased in the high-dose VTX group compared to the control group (Figure 5C,F, respectively). Moreover, the levels of gene and protein expressions of *Tnf-α* (Figure 5D,G, respectively) and *Il-6* (Figure 5E,H, respectively) were remarkably increased in the treatment groups compared to the control group in a dose-dependent manner. Strikingly, the level of the antioxidant protein, Sod-2, significantly declined in rats treated with a high dose of VTX compared to the control group (Figure 5I). Taken together, these results provided important insight regarding the involvement of inflammation and oxidative stress in cardiac toxicity in VTX treatment.

### 2.6. Effect of VTX on Oxidative Stress Status

**Figure 6:**
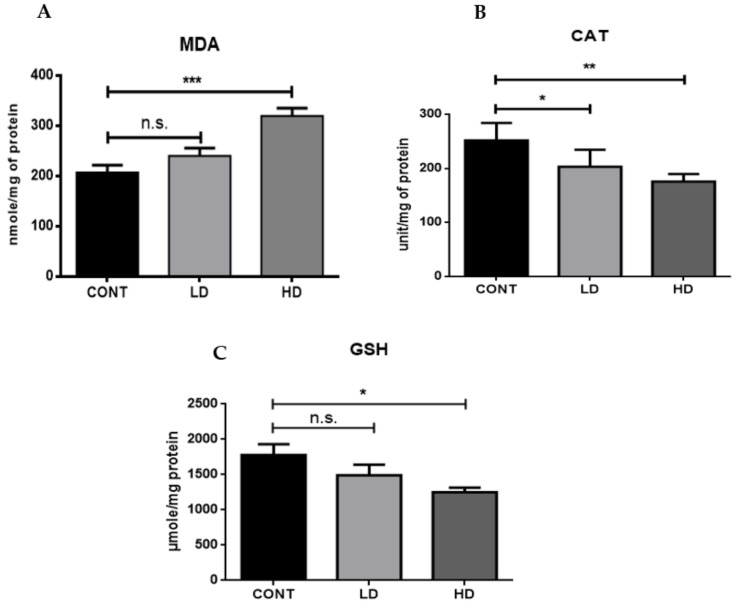
Effects of VTX on oxidative stress status. Biochemical analysis of MDA (**A**), CAT (**B**), and GSH (**C**). Data are presented as mean ± SD (*n* = 5). * *p* < 0.05, ** *p* < 0.01, *** *p* < 0.001; n.s., no significant changes were observed (*p* > 0.05). Cont, control; LD, low-dose venetoclax (50 mg/kg); HD, high-dose venetoclax (100 mg/kg); MDA, malondialdehyde; CAT, catalase; GSH, glutathione.

ROS production is involved in the cardiotoxicity of many anticancer drugs. It can cause oxidative damage to many vital components of the cell, including DNA and proteins, as well as mitochondrial dysfunction and cell death. It had been found that increased levels of lipid peroxidation led to an increase in cardiotoxicity. Moreover, decreased levels of antioxidants in the heart have been associated with the production of ROS and cardiotoxicity. Therefore, we measured the levels of malondialdehyde (MDA), catalase (CAT), and glutathione (GSH). We found that the VTX-treated groups had increased levels of MDA compared to the control group (Figure 6A). Moreover, the levels of CAT (Figure 6B) and GSH (Figure 6C) were remarkably diminished in both VTX-treatment groups compared to the control group. In summary, these results confirmed that VTX treatment induced oxidative stresses.

## 3. Discussion

In the present study, we investigated whether VTX treatment could induce toxic effects in the heart. We found that VTX treatment induced cardiotoxicity that was manifested by changes in the histological architecture of cardiomyocytes, an increase in cardiac enzymes and the expression of relevant genes of cardiac injury, induction of apoptosis markers, alterations in oxidative stress markers, and an increase in inflammatory markers.

VTX is a recently approved anticancer drug that is used for the treatment of multiple hematological cancers [12]. VTX acts as a selective inhibitor of the BH3 domain of Bcl-2 that can restore apoptosis in cancer cells [17]. In the clinical sitting, it has been reported that VTX caused cardiomyopathy and cardiac arrhythmia [19,20,21]. Therefore, we hypothesized that VTX treatment could cause toxic effects to the heart. It is well known that the Bcl-2 family of proteins are essential regulators of apoptosis [22]. Moreover, it has been reported that inhibition of Bcl-2 can lead to apoptosis in different organs, resulting in organ toxicity [21,23]. However, to date, there are no preclinical reports that investigated the cardiotoxicity of VTX. Therefore, in the current study, we investigated the toxic effects of VTX on the heart, and examined the signal and molecular mechanisms of its toxicity.

Cardiac enzymes, such as myocardial muscle creatine kinase (CK-MB) and cardiac troponin (cTn-I), are key tools for evaluating heart damage, as well as histopathological examination of cardiomyocytes, which have been found to be a major marker of the cardiotoxic effect of anticancer drugs [24,25]. Furthermore, alterations in the enzymatic reaction, such as CK-MB and cTn, represent a key early response to toxicant exposure in the heart [26]. In the present study, we found that levels of both CK-MB and troponin I had increased significantly in the VTX-treated rats compared to the nontreated controls. These results indicated that VTX treatment was associated with cardiomyocyte damage. Moreover, our histopathological findings in rats that received VTX treatment showed the presence of minimal nuclear enlargement and focus of myocardial damage associated with a chronic inflammatory reaction. Moreover, we showed that VTX treatment substantially increased the size of cardiac myocytes in a dose-dependent manner. These histopathological findings also confirmed that VTX treatment caused cardiomyocytes injuries.

Cardiac hypertrophy is thought to be a maladaptive compensatory mechanism of the heart in response to toxic insult [27]. Changes in cardiac hypertrophic markers such as *Bnp*, *α-Mhc,* and β-*Mhc* have been found to be associated with cardiac toxicity, and were reported for several anticancer drugs, such as doxorubicin and sunitinib [28,29]. In the current study, we found that VTX treatment increased the gene expression of β-*Mhc*, *Bnp,* and the β*-Mhc:α-Mhc* ratio compared to the control group, while *α-Mhc* gene expression was decreased. These results suggested a VTX-mediated hypertrophy and toxicity on the heart.

Cardiomyocyte death is considered the main cause of cardiotoxicity [30]. The most common form of cell death in drug-induced cardiotoxicity is apoptosis [30,31]. Many cytotoxic insults, such as DNA damage and oxidative stress, can activate the intrinsic apoptotic pathway, which is controlled by the Bcl-2 family of proteins [32,33]. This family can be divided into proapoptotic and antiapoptotic members. The Bcl-2 protein is present in the outer mitochondrial membrane, and acts as an antiapoptotic protein by preventing the release of cytochrome c into the cytosol. On the other hand, intrinsic cell apoptosis can also be regulated by Bax, a proapoptotic member of the Bcl-2 family that can cause cytochrome c release and activate multiple caspases, eventually leading to cell death. Thus, the balance between Bcl-2 and Bax can influence the cell survival or death [23,30,34]. In this study, we found that 21 days of treatment with VTX led to cardiomyocyte death, as evidenced by reduced levels of the gene and protein expressions of Bcl-2, increased levels of *Bax* gene expression, and elevated cleaved caspase-3 (Cleaved Cas-3) protein expression levels.

It is well known that cardiomyocyte death induced by ROS production is involved in many cardiac pathological conditions, such as cardiac hypertrophy and HF [35,36]. Moreover, overproduction of oxygen free radicals and induction of oxidative stress can lead to chemokine production, recruitment of inflammatory cells, and activation of transcription factors such as Nf-κb [36]. Inflammation as a result of oxidative stress is associated with a plethora of pathological diseases, including diabetic cardiomyopathy, congestive cardiomyopathy, and hypertensive heart disease [37]. Increased levels of cytokines in the blood or cardiomyocyte have been found in diseases that lead to cardiomyocyte death. Nf-κb is a transcription factor that upregulates the production of downstream inflammatory mediators, including Tnf-α, Il-6, Ifn-γ, and Tgf-β [38]. Moreover, Ifn-γ and Il-1 are both proinflammatory cytokines that can induce Tnf-α production by cardiomyocytes [38]. Nevertheless, Il-6, which is a proinflammatory cytokine, was found to be elevated in patients with heart failure [26]. Tnf-α is one of the inflammatory mediators that has an important role in the induction of myocardial cell apoptosis [26]. Furthermore, Tgf-β plays an important role in apoptosis, wound healing, and immune regulation [39]. It has been reported that Tgf-β overexpression was associated with fibrosis and hypertrophy [40]. Our findings revealed that 21 days of treatment with VTX resulted in ROS production and inflammation that led to apoptosis of cardiomyocytes, which was confirmed by the increase in the gene and protein expression of Nf-κb-p-65 and the decrease in the Sod-2 protein level. Additionally, the induction of *Ifn-γ* gene levels, as well as *Tnf-α* and *Il-6* gene- and protein-expression levels, further confirmed the toxic consequences of VTX in the heart. Lastly, increased *Tgf-*β gene-expression levels confirmed our histopathological findings, in that VTX treatment induced maladaptive cardiac hypertrophy and eventually cardiac damage.

To further confirm the oxidative stress production, we measured the levels of malondialdehyde (MDA), catalase (CAT), and glutathione (GSH) activity. MDA is a lipid peroxidation end-product and a gold standard marker of lipid peroxidation and oxidative stress. Moreover, GSH and CAT are key endogenous antioxidants that are critical to maintaining cellular homeostasis and ROS levels in response to different toxic insults [41,42]. In this study, we observed a significant dose-dependent reduction in GSH and CAT in response to VTX treatment. Furthermore, our results also demonstrated a significant dose-dependent increase in MDA levels in the VTX-treated groups compared to the control group. These results confirmed our previous findings that VTX treatment induced oxidative stress in the heart.

## 4. Materials and Methods

### 4.1. Animals

Male Sprague-Dawley rats weighing 180–200 g were used. The rats were obtained from Prince Naif Bin AbdulAziz Health Research Center, King Saud University, Riyadh, Saudi Arabia. All experiments on rats were conducted according to the standard guidelines and approved by the Animal Care and Use Committee at King Saud University, Saudi Arabia (Approval# KSU-SE-19-86). Animals were housed under normal laboratory conditions (temperature of 25 ± 1 °C) of a 12 h light/dark cycle with free access to water and a normal chow diet.

### 4.2. Study Design

Rats were arbitrarily divided into three groups, with 8 rats in each group. These groups were as follows:

**Group 1:** Rats in this group were treated daily via oral gavage with 0.9% NaCl for 21 days, and it served as the control group.

**Group 2:** Rats in this group were treated daily with a low dose of VTX (50 mg/kg) via oral gavage for 21 days (LDV).

**Group 3:** Rats in this group were treated daily with a high dose of VTX (100 mg/kg) via oral gavage for 21 days (HDV).

VTX doses were selected based on previous published studies [43,44,45,46,47]. For all groups, rats were weighed daily to calculate the dose and monitored for any changes in weight, as well as for any signs of toxicity. On day 21, rats were anesthetized using ketamine 100 mg/kg and xylazine 10 mg/kg intraperitoneally [48]. Blood samples were collected from all rats. Then, the serum was separated for further measurement of cardiac enzymes. Consequently, heart tissues were harvested and washed twice with ice-cold phosphate-buffered saline; some tissues were fixed in formaldehyde solution (4%) for histopathology studies, while other tissues were stored at −80 °C to conduct different biochemical, gene, and protein studies. The ratio of heart weight to body weight (HW/BW) was used as an indicator of myocardial mass, as described previously [49,50].

### 4.3. Measuring the Cardiac Enzymes

Coagulated blood was centrifuged at 2000× *g* for 10 min at 4 °C to separate the serum from the whole blood. Serum creatine kinase MB isoenzyme (CK-MB) and cardiac troponin I (cTn-I) were measured using a commercially available enzyme-linked immunosorbent assay (ELISA) (ABBEXA, Cambridge, UK) as per the manufacturer’s protocol.

### 4.4. Gene Expression Studies

Total cellular RNA was isolated from the heart tissues by using TRIzol reagent (Ambion, Austin, TX, USA) following the manufacturer’s protocol. The isolated RNA was assessed by using a NanoDrop™ 8000 spectrophotometer (ThermoFisher Scientific, Waltham, MA, USA) to verify the quality and quantity. Then, the isolated RNA (1 µg) was reverse transcribed to cDNA by using a reverse transcription kit (BIMAKE, Houston, TX, USA). Gene expressions were quantified by using the appropriate primers listed in Table 1, a 7500 Fast Real-Time PCR system (ThermoFisher Scientific, Waltham, MA, USA), and SYBR green master mix (BIMAKE, Houston, TX, USA). Data were normalized to β-actin as a housekeeping gene. The forward and reverse primers used are listed in Table 1. The data were acquired during the extension step. All primers were obtained from Integrated DNA Technologies (IDT, Leuven, Belgium). The DDCt method was used to calculate the relative levels of mRNA expression [51].

### 4.5. Protein-Expression Studies

A Western blot was used to determine the protein levels of Nf-κb-p-65, Sod-2, Cleaved Cas-3, Bcl-2, Tnf-α, and Il-6. In brief, previously collected heart tissues were homogenized using Dounce homogenizer in ice-cold RIPA lysis buffer (ThermoFisher Scientific, Waltham, MA, USA) that was supplemented with protease and phosphatase cocktail inhibitor (ThermoFisher). After that, an equal amount of proteins (25–50 µg) were electrophoresed using SDS-PAGE, then transferred onto a PVDF membrane. Following the transfer, membranes were blocked for one hour at room temperature with 5% nonfat dry milk with gentle rocking. Membranes were incubated overnight in the primary antibody at 4 °C with gentle rocking (dilution at 1:1000). These primary antibodies were rabbit anti-nuclear factor kappa B-p-65 (Nf-κb-p-65) antibody, rabbit anti-superoxide dismutase-2 (Sod-2) antibody, rabbit anti-cleaved caspase-3 (Cleaved Cas-3) antibody, rabbit anti-B-cell lymphoma-2 (Bcl-2) antibody, rabbit anti-tumor necrosis factor (Tnf-α) antibody, rabbit anti-interleukin 6 (Il-6) antibody, and mouse anti-B-actin antibody. Thereafter, membranes were incubated for one hour at room temperature with the appropriate horseradish peroxidase (HRB) conjugated secondary antibody (dilution at 1:5000) (ABclonal, Wuhan China). Finally, membranes were visualized using chemiluminescence reagent (Millipore, Burlington, MA, USA) and imaged using a Bio-Rad gel-imaging system (Bio-Rad, Hercules, CA, USA).

### 4.6. Measurement of Lipid Peroxidation

Lipid peroxidation was measured in cardiac tissues by adding thiobarbituric acid (TBA) and trichloroacetic acid (TCA) to tissue homogenates. Then, this mixture was incubated for 30 min in a shaking water bath at 90 °C. Then, the samples were placed on ice for 10 min. Thereafter, the samples were centrifuged for 15 min at 3000× *g* in a refrigerated centrifuge, and the supernatant was measured at 540 nm. The values of results were expressed in nmol of MDA formed per mg of protein [52].

### 4.7. Measurement of Reduced Glutathione

The amount of GSH in tissues was measured using a previously described method [53]. In brief, 5,50-dithio bis (3-nitrobenzoic acid) was added to the reaction mixture. Then, the absorbance was immediately recorded at 412 nm. The values of GSH were expressed as nmol/mg of protein.

### 4.8. Measurement of Catalase Activity

The postmitochondrial supernatant (PMS) from heart tissue was used to estimate the CAT activity by using a previously described method [54]. In brief, the reaction mixture, in a total volume of 3 mL, consisted of 1.95 mL (0.1 M, pH 7.4) phosphate buffer, 1 mL (0.019 M) hydrogen peroxide, and 0.05 mL PMS. The absorbance was recorded for 5 min at 240 nm at an interval of 1 min. To calculate the activity of CAT, the difference in the absorbance was used as the amount of moles of H2O2 changed per min per mg of protein.

### 4.9. Histopathology

Heart tissues from all groups were collected, fixed in 4% formaldehyde, and embedded in paraffin. Then, thin 3 mm sections were prepared using a microtome and stained with hematoxylin and eosin (H&E) to examine the heart morphology. The morphology of the cardiac cells and the nucleus of myocardial fiber cells were compared using an optical microscope to evaluate the severity of cardiac damage (Olympus BX microscope and DP72 camera, Melville, FL, USA). The damage quantification from at least 10 areas corresponding to the myocardial tissue was graded using the following parameters: nuclear enlargement and inflammation based on a four-score evaluation system (0, histopathological changes = 1–25%; 1, histopathological changes = 26–50%; 2, histopathological changes = 51–75%; and 3, histopathological changes =76–100%). This procedure was conducted in at least 10 random areas in each heart section, in three animals from each group, at 400× magnification. The mean score for each parameter was calculated and subjected to statistical analysis. The cardiomyocyte cross-sectional size was estimated and evaluated using the H&E stained slides.

### 4.10. Statistical Analysis

All data are presented as mean ± SD and analyzed using GraphPad Prism version 6.01 (San Diego, CA, USA). Different results between groups were analyzed using a one-way or two-way analysis of variance (ANOVA), followed by a Tukey–Kramer multiple-comparisons test with significance values of *p* < 0.05.

## 5. Conclusions

**Figure 7:**
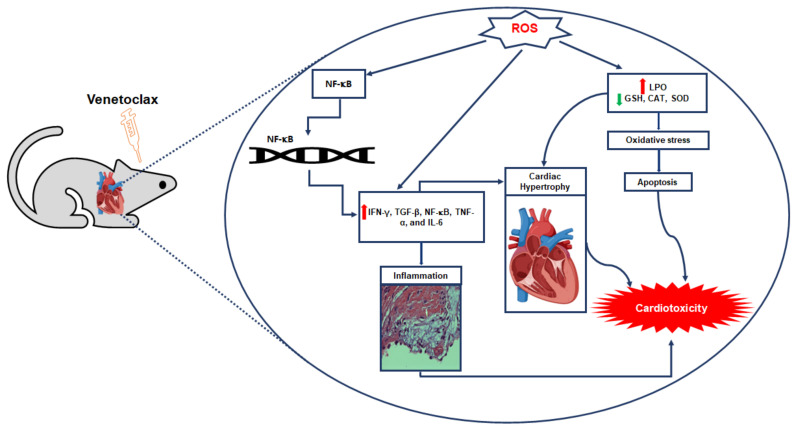
Schematic representation of cardiotoxicity mechanism of venetoclax (VTX).

To the best of our knowledge, this was the first study to report that VTX treatment could induce cardiotoxicity in a dose-dependent manner. This cardiotoxicity caused by VTX treatment was manifested in different ways, including modification or changes in the histological architecture of cardiomyocytes, increases in cardiac enzymes such as CK-MB and troponin I, and alteration of cardiac hypertrophic genes markers such as *Bnp*, *α-Mhc,* and β-*Mhc* (Figure 7). Our findings revealed that VTX treatment induced apoptosis in cardiac tissues as a result of Bcl-2 reduction and Cleaved Cas-3 and Bax induction. Moreover, increased levels of MDA in cardiac tissues and reduced levels of GSH, CAT, and Sod-2 caused oxidative stress that led to activation of Nf-κb-p-65 and induction of the inflammatory response, which was observed as the increases in the expressions of Ifn-γ Tgf-β, Tnf-α, and Il-6. The results of this study add to the current knowledge regarding the safe use of VTX. One of the limitations of the present study was that we did not use knockout models to inhibit the molecular mechanisms involved in VTX-induced cardiotoxicity. Furthermore, we did not measure any in vivo parameters, such as those from echocardiography or blood pressure measurements, which could help to further assess the effects of VTX on heart function. However, the results of the current study shed light on the toxic effects of VTX treatment on the heart, and encourage future studies to further prove the current findings. Further studies are required to fully and comprehensively understand the exact mechanism of VTX-induced cardiotoxicity.

## Figures and Tables

**Table 1:** List of forward and reverse primers for different genes used in the qRT PCR analysis.

Gene	Primer Sequence (5′-3′)	Product Length (bp)	Accession Number
***Bcl-2***	Forward: CATGCGACCTCTGTTTGA Reverse: GTTTCATGGTCCATCCTTG	193	NM_016993.2
***Bnp***	Forward: CAGAAGCTGCTGGAGCTGATAAG Reverse: TGTAGGGCCTTGGTCCTTTG	78	NM_031545.1
***Tnf-α***	Forward: CACGCTCTTCTGTCTACTGA Reverse: GTACCACCAGTTGGTTGTCT	254	NM_012675.3
***Il-6***	Forward: GCCCTTCAGGAACAGCTATGA Reverse: TGTCAACAACATCAGTCCCAAGA	80	NM_012589.2
***α -Mhc***	Forward: TGAAGAGCGCAGAGACAGAGAA Reverse: TTCTCCTCTGCGTTCCTACACT	2743	NM_017239.2
**β -*Mhc***	Forward: GAACCCTCCCAAGTTCGACAAGATCG Reverse: TGTTTCAAAGGCTCCAGGTCTCAGG	5635	NM_017240.2
***Nf-κb-p-65***	Forward: CATGCGTTTCCGTTACAAGTGCGA Reverse: TGGGTGCGTCTTAGTGGTATCTGT	85	NM_199267.2
***Tgf-β***	Forward: GCCTCCGCATCCCACCTTTG Reverse: GCGGGTGACTTCTTTGGCGT	396	NM_021578.2
***Inf-γ***	Forward: ATGAGTGCTACACGCCGCGTCTTGG Reverse: GAGTTCATTGACAGCTTTGTGCTGG	405	NM_138880.3
***Bax***	Forward: TAGCAAACTGGTGCTCAAGG Reverse: TCTTGGATCCAGACAAGCAG	111	NM_017059.2
***β -Actin***	Forward: AGTTCGCCATGGATGACGATATCGC Reverse: TGTAAAACGCAGCTCAGTAACAGTCCG	1164	NM_031144.3

## Data Availability

Data are contained within the article.
